# Strategies to develop climate-resilient chili peppers: transcription factor optimization through genome editing

**DOI:** 10.1007/s00425-025-04747-5

**Published:** 2025-06-17

**Authors:** Mallesham Bulle, Md. Mezanur Rahman, Md. Robyul Islam, Sadanandam Abbagani

**Affiliations:** 1 Plant Biotechnology Research Unit, Department of Biotechnology, Kakatiya University, Warangal, Telangana 506 009 India; 2 School of Plant, Environmental, and Soil Sciences, Louisiana State University Agricultural Center, Baton Rouge, LA 70803 USA; 3 Institute of Genomics for Crop Abiotic Stress Tolerance, Department of Plant and Soil Science, Texas Tech University, Lubbock, TX USA; 4 Department of Agroforestry and Environment, Gazipur Agricultural University, Gazipur, 1706 Bangladesh; 5 Institute of Biotechnology and Genetic Engineering, Gazipur Agricultural University, Gazipur, 1706 Bangladesh

**Keywords:** CRISPR/Cas9, *Capsicum* stress tolerance, Genome editing, Multi-omics, Transcription factors

## Abstract

Chili peppers (*Capsicum* spp.), a globally significant crop revered for their nutritional, economic, and cultural importance, are increasingly imperiled by the converging burdens of climate-induced abiotic stresses, including drought, heat, and salinity, and relentless biotic assaults from pathogens and insect herbivores. These overlapping stressors not only destabilize yield but also compromise the metabolic intricacy responsible for the accumulation of health-promoting secondary metabolites. Although *Capsicum* exhibits remarkable genetic and phytochemical diversity, the integrated transcriptional, metabolic, and epigenetic frameworks that underpin its stress resilience remain poorly delineated. This review synthesizes recent advances in decoding core transcription factor families, such as CaNAC, CaWRKY, and CaMYB, that serve as pivotal regulators of osmotic adjustment, reactive oxygen species detoxification, hormonal crosstalk, and secondary metabolite biosynthesis under stress conditions. We further highlight how multi-omics-guided gene discovery, when paired with CRISPR/Cas-mediated genome editing, enables precise reprogramming of key regulatory loci to enhance adaptive responses. Emerging innovations, including base editing, prime editing, and novel nucleases like Cas12a and Cas13d, are expanding the functional genome-editing landscape, while the integration of morphogenic regulators and genotype-independent transformation platforms is beginning to circumvent long-standing obstacles in *Capsicum* genetic engineering. Lastly, we propose a transformative framework that converges transcription factor modulation, multi-omics strategies, precision phenotyping, and next-generation genome editing to accelerate the development of climate-resilient *Capsicum* cultivars with optimized metabolic traits. This strategic convergence of molecular insight and biotechnological innovation offers a robust foundation for building next-generation chili pepper varieties capable of withstanding intensifying environmental and pathogenic pressures, ultimately safeguarding yield, nutritional quality, and agricultural sustainability in the face of global climate change.

## Introduction

Chili pepper (*Capsicum annuum*), a cornerstone Solanaceous crop, thrives in warm climates and is cultivated extensively across Asia, the Americas, Europe, and Africa (Tripodi et al. 2021). This broad ecological distribution underpins its global agricultural prominence, with 2022 yields reaching 37-million tonnes (MT) of fresh peppers and 4.9 MT of dry peppers (FAOSTAT 2023). China led fresh green chili production (16.8 MT), followed by Mexico (3.11 MT) and Indonesia (3.02 MT), while India dominated dry chili output with 1.87 MT (FAOSTAT 2023). The worldwide demand for chili peppers is driven not only by their wide adaptability but also by the high value of their fruits, which are renowned for their vivid color, pungent flavor, and culinary versatility. These traits arise from an array of specialized metabolites, including carotenoids, vitamins C and E, flavonoids, and capsaicinoids, that define both fruit quality and their health-promoting properties (Islam et al. 2023b).

Paradoxically, the core attributes that define the agronomic and economic value of chili pepper are among the most vulnerable to escalating environmental stresses. A wide range of abiotic and biotic stressors, including heat, drought, salinity, pathogens, and herbivores, disrupt cellular, physiologic, and biochemical balance, ultimately compromising fruit quality and causing significant yield losses (Vijay et al. 2023; Islam et al. 2023a; Zhang et al. 2024b). Addressing such challenges requires mechanistic insight into the molecular underpinnings of stress resilience. Transcription factors (TFs), as master regulators of gene expression, play a critical role in orchestrating plant responses to environmental stimuli (Song et al. 2016). Recent advances in CRISPR–Cas genome editing now enable precise manipulation of these TF activity, offering a powerful means to rewire stress-responsive networks with unprecedented specificity (Singha et al. 2022). This strategy enables the targeted enhancement of tolerance traits under complex stress conditions, such as the combination of drought and pathogen attack, while preserving yield stability (Das et al. 2022). Leveraging such tools in chili pepper breeding could accelerate the development of climate-resilient cultivars and redefine future crop improvement pipelines. Achieving this, however, requires a comprehensive understanding of transcriptional regulatory networks and their linkage to measurable stress-adaptive phenotypes.

In response to environmental stress, chili peppers deploy a suite of physiologic and biochemical strategies, including reduced leaf expansion, wilting, leaf abscission, enhanced root growth, and alterations in relative water content (RWC) and membrane stability, to maintain homeostasis and sustain productivity (Salaria et al. 2023; Poudyal et al. 2024). These physiologic responses are often accompanied by elevated production of reactive oxygen species (ROS), reactive nitrogen species (RNS), and free radicals, which, while integral to signaling, can trigger lipid peroxidation, membrane disruption, enzyme inactivation, and cellular damage when not tightly regulated (Rahman et al. 2024). A central mediator of abiotic stress adaptation is abscisic acid (ABA), which governs stomatal closure to limit transpirational water loss and maintain water-use efficiency (WUE) under adverse conditions (Trejo-Paniagua et al. 2024; Bulle et al. 2024b; Zhang et al. 2024b). These responses are coordinated by transcriptional regulatory networks that integrate hormonal and environmental signals into precise gene expression programs.

**Fig. 1:**
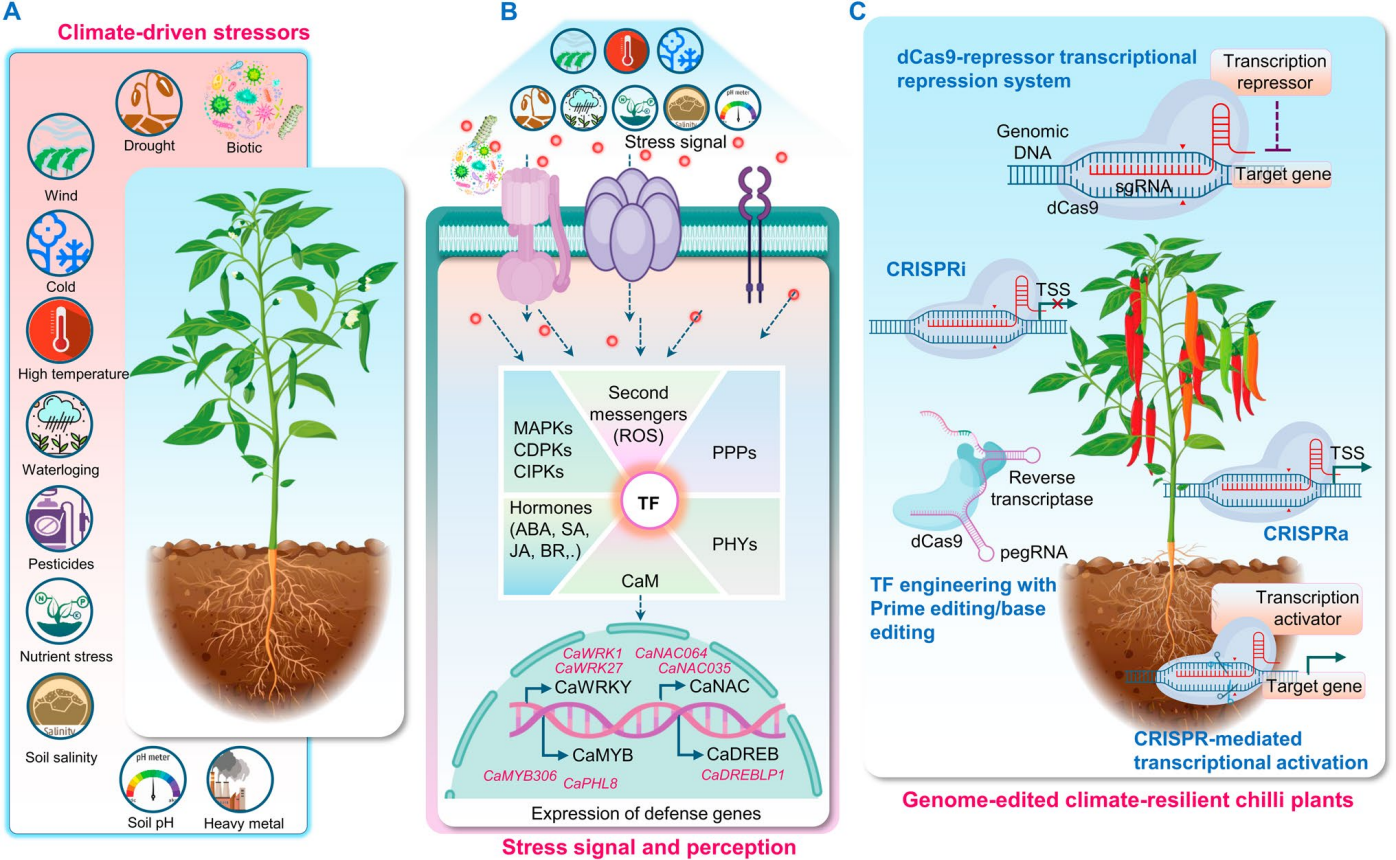
Transcription factor engineering and genome editing strategies for developing climate-resilient chili plants. **A** Chili peppers (*Capsicum spp.*) are routinely challenged by an array of biotic (pathogens, insects, and herbivores) and abiotic (heat, cold, flooding, drought, and imbalance of nutrients or salts) stresses that threaten their growth and productivity. Despite these adversities, chili peppers deploy intricate molecular networks orchestrated by signaling cascades to mount robust stress responses. **B** Stress resilience begins with the detection of external signals by receptors or sensors located on the cell wall or plasma membrane. These sensors translate external stimuli into intracellular signals, initiating a cascade of events that amplify and relay the signal internally. This process often engages reactive oxygen species (ROS), calcium ions (Ca^2+^), and phytohormones such as abscisic acid (ABA), jasmonic acid (JA), salicylic acid (SA), and ethylene (ETH), which serve as secondary messengers. Signal transduction pathways are further modulated by protein kinases (PKs) and phosphatases (PPPs), with two central cascades—the mitogen-activated protein kinase (MAPK) and calcium-dependent protein kinase (CDPK) pathways—playing pivotal roles in stress signaling. These cascades ultimately converge on transcription factors (TFs), the master regulators of gene expression. Activated TFs bind to *cis*-regulatory elements (CREs) within promoter regions of stress-responsive genes, driving transcriptional reprogramming. Key TF families such as CabZIP, CaMYB, CaNAC, CaWRKY, and CaDREB coordinate stress adaptation by modulating genes involved in osmotic balance, ROS detoxification, and other protective mechanisms. **C** This molecular choreography culminates in the expression of stress-resilient phenotypes, bolstering the plant’s capacity to withstand environmental pressures. Through these transcriptional signaling networks, coupled with emerging dCas9-based regulatory systems such as CRISPRko (knock out), CRISPRi (interference), and CRISRPa (activation), chili peppers exemplify the remarkable plasticity of plant stress adaptation, underscoring the potential for biotechnological innovations to further enhance resilience. *PM* plasma membrane, *TFs* Transcription factors, *CRE* *cis*-regulatory element, *Pol II* RNA polymerase II, *bZIP* Basic Leucine Zipper, *MYB* myeloblastosis viral oncogene homolog, *NAC/WRKY* NAM, ATAF1/2, and CUC2, *DREB* Dehydration responsive element-binding, *CRISPR/Cas* Clustered Regularly Interspaced Short Palindromic Repeats/CRISPR-associated protein

At a deeper molecular level, stress adaptation depends on the activation of intricate gene networks that drive protective physiologic and biochemical adjustments (De Nadal et al. 2011; Song et al. 2016; VanWallendael et al. 2019). These genes encode both metabolic proteins, involved in osmolyte production, membrane stabilization, and ROS detoxification, and regulatory proteins that modulate stress signaling pathways (Templeton and Lamb 1988; Shabala et al. 2016; Cao et al. 2023) (Fig. 1). Transcriptomic analyses broadly classify these genes into two functional groups. The first includes genes encoding protective enzymes and structural proteins, such as those involved in the biosynthesis of compatible solutes (e.g., proline, betaine, and soluble sugars), aquaporins, ROS-scavenging enzymes (including glutathione *S*-transferase, catalase, superoxide dismutase, ascorbate peroxidase), and macromolecule-stabilizing proteins such as LEA proteins, osmotin, and molecular chaperones (Xiong et al. 2002; Shahzad et al. 2021; Rahman et al. 2021; Blanc-Mathieu et al. 2024). The second group comprises core regulatory components, primarily TFs, that orchestrate gene expression dynamics under stress. Key families such as NAC (NAM (No Apical Meristem), ATAF1/ATAF2 (*Arabidopsis thaliana* Activating Factor 1/2), and CUC2 (Cup-shaped Cotyledon 2), WRKY (WRKYGQK motif containing TFs), MYB (v-Myb myeloblastosis viral oncogene homolog), bZIP (basic leucine zipper), and DREB (dehydration-responsive element binding factor), all of which function as central hubs in this transcriptional reprogramming. These TFs operate in concert with signaling molecules, including protein kinases (e.g., MAPKs (Mitogen-activated protein kinases), CDPKs (Calcium-dependent protein kinases), receptor-like kinases, and protein phosphatases (PPPs), to propagate and fine-tune stress signaling cascades (Xiong et al. 2002; Shahzad et al. 2021; Blanc-Mathieu et al. 2024) (Fig. 1). Collectively, these regulatory networks underpin the molecular framework underlying stress resilience in plants.

In *Capsicum annuum*, the reference genome spans 3.44 Gb and encodes 34,476 protein-coding genes, including 2153 TFs classified into 80 gene families (Kim et al. 2014; Qin et al. 2014), providing a rich genetic reservoir for targeted functional studies and genome editing. Of these, approximately 511 TFs are associated with biotic and abiotic stress responses, DNA-binding complex formation, and developmental regulation (Qin et al. 2014). These TFs recognize specific *cis*-regulatory elements (CREs) within promoter regions, to active downstream genes that enhance stress resilience (Shahzad et al. 2021). Transcriptomic analysis reveal a densely interconnected regulatory architecture that controls stress tolerance at the transcriptional level (Ma et al. 2023). Yet, how TFs coordinate the integration of multiple signaling pathways, particularly under concurrent biotic and abiotic stresses, remains poorly resolved. Addressing this knowledge gap is critical for decoding stress response crosstalk and accelerating the development of climate-resilient *Capsicum* cultivars.

To address this unresolved complexity, we integrate recent transcriptomic insights with emerging genome editing strategies to outline a transformative roadmap for chili pepper improvement. This review bridges high-resolution transcriptomic data with CRISPR-based innovations, with a focus on TFs as central regulators of stress adaptation. We elucidate how these TFs modulate gene expression, integrate hormonal and environmental cues, and serve as actionable targets for genome editing. By identifying key TFs implicated in both abiotic and biotic stress responses, we highlight precise molecular entry points for CRISPR-mediated intervention. Furthermore, we explore how genome editing technologies can be harnessed to reconfigure stress-responsive regulatory networks, ultimately enhancing resilience under dynamic climatic conditions. By synthesizing current advances and illuminating persistent knowledge gaps, this review provides both a conceptual framework and a practical blueprint for next-generation innovations in *Capsicum* biotechnology. We present a novel synthesis of regulatory genomics and precision editing aimed at unlocking the untapped potential of chili pepper in the face of environmental uncertainty.

### Engineering resilient and metabolite-rich *Capsicum* through transcription factor targeting

Domesticated over 9000 years ago, *Capsicum* species emerged as one of the most genetically diverse horticultural lineages, well-adapted to a spectrum of environmental extremes (Luna-Ruiz et al. 2018; MacNeish 1964; Duranova et al. 2022; Liu et al. 2023). Beyond their culinary and cultural relevance, selected species, including *C. annuum*, *C. baccatum*, *C. chinense*, *C. frutescens*, *C. pubescens*, and *C. assamicum*, harbor a rich repertoire of secondary metabolites with pharmacological and ecological significance (Ramchiary and Kole 2019; Liu et al. 2023).

**Fig. 2:**
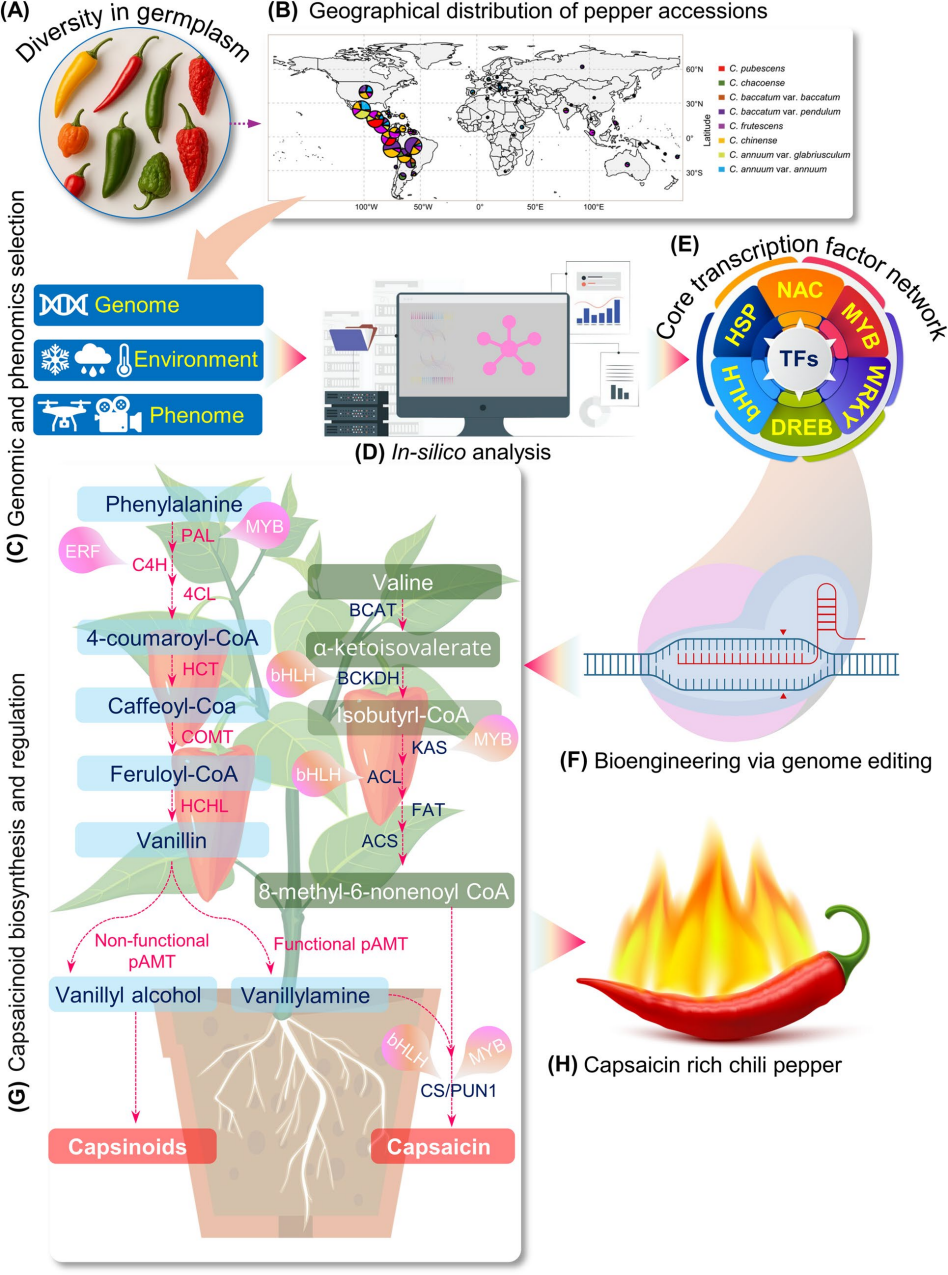
Schematic illustration for engineering climate-smart and metabolite-rich *Capsicum.* **A**, **B** The Cultivated *Capsicum* species exhibit extensive morphologic and agronomic diversity, primarily in Central and South America (Liu et al. 2023). The landraces are great sources for conservation and breeding programs. Future *Capsicum* improvement requires a comprehensive understanding of its biology, extending beyond individual traits to a system-level perspective. **C** The genomic and phenomics selection process will provide the foundation for molecular design, **D** whereas *in-silico* approaches facilitate the application of stress-resilient alleles and genes, aiding in the dissection of gene networks that regulate responses to diverse environmental factors, including biotic and abiotic stresses, and metabolite content of chili cultivars. **E** Targeting transcription factors (TFs) is one of the key strategies for climate-smart and nutrient-rich chili peppers. Rewiring of TFs also contributes to the modulation of major secondary metabolites in *Capsicum.* **F** CRISPR*-*mediated modification of TFs will enable the precise engineering of these traits at the genetic and molecular level. **G** The TFs involved in the capsaicinoids biosynthesis pathway (Islam et al. 2023b) have great potential for future CRISPR targets for **H** capsaicin improvement. Pinkish-colored baubles represent various transcription factors known to regulate genes involved in capsaicin biosynthesis and accumulation. Dashed magenta arrows indicate the enzymatic steps of shikimate and fatty acid metabolic pathways. *PAL*- Phenylalanine ammonia lyase, *C4H*-Cinnamate 4-hydroxylase, *4CL*−4-Coumaroyl-CoA ligase, *HCT* Hydroxycinnamoyl transferase, *COMT*-Caffeoyl-CoA O-methyltransferase, *pAMT*-Putative aminotransferase, and CS/PUN1-Capsaicin Synthase/Pun1 (AT3) Acyl transferase). Transcription factor genes such as *ERF* Ethylene Responsive Factor, *MYB*-v-Myb myeloblastosis viral oncogene homolog, *bHLH*-basic helix-loop-helix, *BCAT*-Branched-chain amino acid transferase, *BCKDH*-Branched-chain α-ketoacid dehydrogenase, *ACS*-Acyl-CoA synthetase, *KAS*-Ketoacyl-*ACP* Synthase, *ACL*-Acyl carrier protein, *FAT*-Fatty acid thioesterase

Among specialized metabolites, capsaicin stands out as a hallmark compound that not only imparts pungency but also exemplifies stress-inducible biosynthetic pathway (Islam et al. 2023b). Its production involves the convergence of the phenylpropanoid and branched-chain fatty acid pathways, generating vanillylamine and 8-methyl-6-nonenoic acid, respectively. Key enzymes in these routes include phenylalanine ammonia-lyase (PAL), caffeic acid O-methyltransferase (COMT), ketoacyl-ACP synthases I and III (KAS I/III), acyl carrier protein (ACL), acyl-CoA synthetase (ACS), ketoacyl-ACP reductase (CaKR1), and acyl-ACP thioesterase (FatA). These precursors are ultimately condensed by capsaicin synthase, encoded by *Pun1* (also known as *AT3*), to form capsaicin (Arce-Rodríguez and Ochoa-Alejo 2019) (Fig. 2). Genomic studies in *Capsicum chinense* have identified 58 structural genes associated with capsaicin biosynthesis. Among them, *CcPun1* (*Pungency gene 1*), *CcpAMT-2* (*putative aminotransferase-2*), and *CcCOMT10* (*caffeic acid O-methyltransferase 10*) exhibit peak expression between 30 and 50 days after pollination. Notably, a loss-of-function mutation in *pAMT*, which catalyzes the formation of vanillylamine, results in the accumulation of capsinoids—non-pungent analogs of capsaicin—commonly observed in sweet cultivars such as CH-19 (Zhang et al. 2022b).

Transcriptional regulation of capsaicinoid biosynthesis is orchestrated by MYB31, a master regulator that directly activates multiple structural genes in the pathway (Arce-Rodríguez and Ochoa-Alejo 2017) (Fig. 2). Loss-of-function mutations in MYB31 are associated with non-pungent phenotypes, while promoter variants in *C. chinense* enhance its transcriptional activity through the recruitment of WRKY9, amplifying downstream gene expression (Arce-Rodríguez and Ochoa-Alejo 2017; Han et al. 2019; Zhu et al. 2019). Several bHLH (basic helix-loop-helix) transcription factors—including CabHLH07, CabHLH09, and CabHLH026—physically interact with MYB31, modulating metabolic flux, particularly under stress conditions (Liu et al. 2021a). Additional regulators, such as the MeJA-responsive CaMYB108, ethylene-responsive factors CcERF2, CaERF53, and CaERF66, and newly identified MYB TFs including CcMYB48 and CaMYB37, further fine-tune the pathway through promoter binding and transcriptional co-expression (Sun et al. 2019; Song et al. 2020; Islam et al. 2021, 2023a, 2024; Arce-Rodríguez et al. 2021; Wen et al. 2022). Natural allelic variation among these regulatory genes adds yet another layer of control, shaping pungency diversity across *Capsicum* cultivars. More fundamentally, abiotic and biotic stresses profoundly reshape secondary metabolism in *Capsicum*, frequently acting as key triggers for capsaicinoid biosynthesis. Environmental cues such as drought, elevated temperatures, and pathogen attack modulate the expression of core regulatory transcription factors—including MYB31, WRKY9, and various stress-inducible ERFs—thereby activating downstream biosynthetic genes (Fig. 2) (Islam et al. 2023b). These stress signals interact with hormonal pathways involving jasmonates, salicylic acid, and ethylene, resulting in either amplification or suppression of metabolite accumulation. This dynamic regulatory network underscores the ecological role of capsaicinoids not only as chemical deterrents against herbivores and pathogens, but also as adaptive metabolites that enhance plant resilience under fluctuating environmental conditions (Fig. 1).

Recent multi-omics approaches are shedding light on the regulatory circuits underpinning *Capsicum* stress adaptation. Integrated transcriptomic and metabolomic analyses of heat-tolerant (17CL30) and heat-sensitive (05S180) cultivars identified over 5700 differentially expressed genes and more than 100 differentially accumulated metabolites, with glutathione metabolism emerging as a key axis of thermotolerance (Wang et al. 2019). Parallel studies under cold and salinity stress have revealed conserved transcriptional responses related to osmoprotection, membrane stabilization, and ion homeostasis (Gao et al. 2022; Zhang et al. 2022a). Across these conditions, TFs from the DREB, NAC, WRKY, bZIP, and MYB families consistently emerge as regulatory hubs (Fig. 1) (Table 1), underscoring their potential as high-leverage targets for genome-guided reprogramming. Beyond primary metabolic regulation, transcriptional control of specialized metabolism reinforces biochemical resilience, while structural and immunologic adaptations offer additional, and often synergistic, avenues of stress mitigation. Traits such as trichome architecture and inducible disease resistance are likewise governed by transcriptional networks—and represent untapped targets for genome editing strategies aimed at fortifying the plant’s frontline defense (Kabir et al. 2024). Trichomes serve as the plant’s initial line of defense, modulating interactions with both abiotic stressors and biologic antagonists (Johnson and Baucom 2024). In *Capsicum*, comparative transcriptomic analyses between hairy (GZZY-23) and glabrous (PI246331) genotypes revealed the upregulation of TFs associated with epidermal cell differentiation, secondary metabolism, and hormonal crosstalk—particularly MYB, bHLH, HD-Zip (Homeodomain-leucine zipper), and zinc finger proteins (Baillo et al. 2019; Hu et al. 2022; Shen et al. 2024). Crosstalk between auxin and jasmonic acid (JA) signaling pathways appears integral to trichome patterning and elongation (Gao et al. 2021), highlighting a complex hormonal-transcriptional interface amenable for precise genetic modulation.

**Table 1:** Potential transcription factor gene(s) to improve chili peppers by employing CRISPR genome editing

Family	Gene	Gene induction condition	Subcellular localization	Functional validation	Associated stress responses	References
NAC	*CaNAC035*	LT, HT, OS, Salt, GA, MeJA, SA, and ABA	Nucleus	Pepper and *Arabidopsis*	Cold, HT, NaCl, and mannitol	Zhang et al. (2020b)
		Cold and ABA		Pepper	Cold	Zhang et al. (2024a)
	*CaNAC064*	4 °C, 40 °C, SA and ABA	Nucleus	Pepper and *Arabidopsis*	Cold	Hou et al. (2020)
	*CaNAC46*	HT, cold, high salt, drought, ABA, SA, and MeJA	Nucleus	Pepper and *Arabidopsis*	Drought and salt	Ma et al. (2021)
	*CaNAC2*	Cold, salt and ABA	Nucleus	Pepper	Chilling and Salt	Guo et al. (2015b)
	*CaNAC2c*	HT and *Ralstonia solanacearum* infection	Nucleus	Pepper and Tobacco	Pathogen (*R. solanacearum*) and high temperature	Cai et al. (2021)
	*CaNAC1*	Cold	Nucleus	Pepper	Cold	Kong et al. (2020)
MYB	*CaMYB306*	Cold and fruit coloration	Nucleus	Pepper and Tomato	Cold	Ma et al. (2022)
	*CaDIM1* (*Capsicum annuum* Drought Induced MYB 1)	ABA and drought	Nucleus	Pepper and *Arabidopsis*	Drought	Lim et al. (2022)
	*CaPHL8*	Pathogen (*R. solanacerum*)	Nucleus	Pepper	Pathogen	Noman et al.(2019)
WRKY	*CaWRKY1*	*Xanthomonas axonopodis, Pseudomonas syringe* and SA	Nucleus	Pepper and Tobacco	Pathoges (*X.axonopodis*, *TMV* and *P. syringe*)	Oh SangKeun et al. (2008)
	*CaWRKY27*	SA, MeJA, ETH and *R. solanacearum*	Nucleus	Pepper and Tobacco	*R.solanacearum* infection	Dang et al. (2014)
		HT and H_2_O_2_	Nucleus	Pepper and *Arabidopsis*	HT	Dang et al. (2018)
	*CaWRKY22*	*R. solanacearum*, SA, MeJA and ETH	Nucleus	Pepper	*R. solanacearum* infection	Hussain et al. (2018)
	*CaWRKY30*	Pathogens (*Meloidogyne incognita*, *Tobacco mosaic virus*, *R. solanacerum*, and *Phytophthora capsici*), and ET, SA, ABA	Nucleus	Pepper	Pathogen stress responses	Jingyuan et al. (2011); Hussain et al. (2021)
	*CaWRKY40*	*R. solanacearum* and heat shock	Nucleus	Pepper and Tobacco	Heat shock and* R*. *solanacearum*	Dang et al. (2013)
	*CaWRKY58*	Pathogen (*R. solanacearu*)	Nucleus	Pepper and Tobacco	Pathogen (*R*. *solanacearum*)	Wang et al. (2013)
	*CaWRKY3*	*R.solanacearum*, SA, MeJA and ETH	N/A	Pepper and *Arabidopsis*	Pathogen (*R*. *solanacearum*)	Hussain et al. (2024)
	*CaWRKY50*	*Colletotrichum scovillei* and SA	Nucleus	Pepper and Tomato	Silencing of *CaWRKY50-* showed enhanced pepper resistance to *C. scovillei*	Li et al. (2023)
	*CaWRKY01-10 and CaWRKY08-4*	Pathogen (*P. capsici*)	Nucleus	Pepper and Tobacco	Pathogen (*P. capsici*)	Cheng et al. (2024)
GRAS	*CaGRAS1*	Drought, high salinity, and ABA	Nucleus and cytoplasm	Pepper and *Arabidopsis*	Drought	Oh et al. (2022)
HSP/HSF	*CaHsfA2*	HT	Nucleus	Pepper	HT	Guo et al. (2015a)
	*CaHsp25.9*	HT, salt and drought	Cell membrane and Cytoplasm	Pepper and *Arabidopsis*	HT, salt and drought	Feng et al. (2019)
	*CaHSP18.1*a	HT, salt and drought	Cell membrane	Pepper and *Arabidopsis*	HT, salt and drought	Liu et al. (2021a, b)
	*CaHsp70-1*	HT, CaCl_2_, H_2_O_2_ and putrescine (Put)	Cytoplasm and Nucleus	Non-transgenic approach to compare B6, pepper thermosensitive line; R9, pepper thermotolerant line	HT	Guo et al. (2014)
bZIP	*CAbZIP1*	Pathogens (*Xanthomonas campestris* pv. Vesicatoria, and *Pseudomonas fluorescens) *ABA, NaCl, MeJA, SA, and MV	Nucleus	*Arabidopsis*	Pathogen (*Pseudomonas syringae pv. tomato* DC3000), drought and salt	Lee et al. (2006)
	*CabZIP2*	*Xanthomonas campestris pv. Vesicatoria* and MeJA, SA, and ETH	Cytoplasm and Nucleus	*Arabidopsis*	Pathogen (*P. syringae* pv. *Tomato* DC3000)	Lim et al. (2015)
	*CabZIP25*	Abiotic stresses and phytohormones	N/A	Pepper and *Arabidopsis*	Salt	Gai et al. (2020)
DREB	*CaDREBLP1*	Dehydration, high salinity and wounding	Nucleus	Non-transgenic pepper	Water and salt	Hong and Kim (2005)

*NAC-NAM* No Apical Meristem, *ATAF1/ATAF2* *Arabidopsis thaliana* Activating Factor 1/2, and *CUC2* Cup-shaped Cotyledon 2, *MYB*- v-Myb myeloblastosis viral oncogene homolog, *WRKY* WRKYGQK motif containing TFs, *GRAS* Gibberellic Acid Insensitive (GAI), Repressor of GAI (RGA), and Scarecrow-Like (SCL) protein family; HSP/HSF- Heat shock protein/heat shock factor gene family; *bZIP* basic leucine zipper, *DREB* dehydration-responsive element binding factor, *LT* Low temperature, *HT* High temperature; *OS* Osmotic Stress, *GA* Gibberellic Acid, *MeJA* methyl-jasmonic acid, *SA* salicylic acid, *ABA* abscisic acid, *ETH* ethephon, *ET* Ethylene, *CaCl*_*2*_—Calcium dichloride, *H*_*2*_*O*_*2*_ Hydrogen Peroxide, *MV* Methyl viologen

Yet, a mechanistic understanding of the epigenetic underpinnings of trichome plasticity remains elusive. It is plausible that environmentally induced variation in trichome traits is governed not only by TF dynamics but also by chromatin-level regulation (Kabir et al. 2024). High-resolution epigenomic profiling will be essential to elucidate how DNA methylation and histone modifications influence the transcriptional accessibility of key trichome-related loci under fluctuating environmental regimes (Johnson and Baucom 2024; Kabir et al. 2024). Concurrently, disease resistance in *Capsicum* is underpinned by an equally intricate transcriptional framework. Comparative transcriptomics between anthracnose-resistant (IIVRC-452) and susceptible (Pusa Jwala) lines identified over 3000 differentially expressed transcripts, including *CaCYP76A2* (*C. annuum cytochrome P450 76A*2), *CaLAG-1* (*C. annuum late embryogenesis abundant group 1*), *CaLOX* (*C. annuum lipoxygenase*), *CaPG* (*C. annuum Polygalacturonase*), and *CaSAP-13* (*C. annuum stress-associated protein 13*)—genes involved in oxidative stress mitigation and defense signal transduction (Kumar et al. 2022). These immune responses are transcriptionally coordinated and embedded within larger stress-responsive networks. Supporting this, proteomic and metabolomic datasets reveal stress-induced reprogramming of mitochondrial energy metabolism, flavonoid biosynthesis, and redox balance under UV-B and cold conditions (Morales-Merida et al. 2024). Such dynamic adjustments are governed by upstream transcriptional regulators that recalibrate cellular function in response to environmental flux. Together, these findings elevate TF from mere gene switches to central integrators of stress resilience. With key regulatory nodes and defense pathways now illuminated, the stage is set for the strategic deployment of genome editing tools to rewire *Capsicum*’s resilience from the inside out.

### Advancing chili pepper breeding with CRISPR: precision editing for stress resilience and trait improvement

Building on this foundational understanding of transcriptional regulation, the deployment of genome editing technologies, particularly CRISPR/Cas systems, now offers a transformative path to reprogram regulatory hierarchies with unprecedented precision (Fig. 1) (Mishra et al. 2021; Vijay et al. 2023; Bulle et al. 2024a; Choi et al. 2024). These tools enable the strategic manipulation of key transcriptional nodes, allowing multigenic stress tolerance and metabolic enhancement through targeted single-locus modifications. Historically, in chili pepper, traditional breeding approaches—ranging from hybridization to mutation breeding—have introduced valuable traits into elite cultivars, enabling improved adaptation across diverse agroecological zones (Barchenger et al. 2018; Islam et al. 2023a, 2024; Vijay et al. 2023). Yet, these methods are inherently limited by their labor-intensive nature, extended timelines, and the complexity of polygenic traits shaped by gene–environment interactions and pleiotropic effects (Varshney et al. 2021). Advances in molecular biotechnology are now redefining the frontiers of crop improvement. Transgenic approaches, including RNA interference (RNAi) and gene overexpression, have been employed to generate genetically modified (GM) chili peppers with enhanced resistance to pathogens and abiotic stressors (Lim et al. 2015; Bulle et al. 2016, 2024b; Mahto et al. 2020). By directly manipulating stress-responsive genes or defense regulators, researchers have alleviated key vulnerabilities in *Capsicum* germplasm (Momo et al. 2022). However, the deployment of GM crops continues to face formidable regulatory, societal, and commercial barriers, which constrain their widespread adoption (Quemada 2022; Esquivel et al. 2023).

Amid mounting limitations of transgenic interventions, genome editing technologies, particularly CRISPR/Cas9, have emerged as a paradigm-shifting tool for precision crop improvement (Figs. 1 and 2) (Irfan et al. 2024). Unlike conventional genetic modification, CRISPR enables targeted, site-specific alterations within native genomes without the integration of foreign DNA, thereby circumventing many regulatory bottlenecks and mitigating public concerns surrounding transgenics (Zhang et al. 2020a; Gupta et al. 2021). This precision, coupled with its efficiency and versatility, has catalyzed significant advances in enhancing drought resilience, pest resistance, and nutrient-use efficiency across multiple crop species (Haque et al. 2018). In *Capsicum*, the integration of CRISPR-mediated gene editing with genomic selection and high-throughput phenotyping—augmented by artificial intelligence—is redefining the pace and resolution of breeding pipelines (Barchenger et al. 2018; Momo et al. 2022; Islam et al. 2023a).

The CRISPR toolkit has further expanded with the characterization of alternative nucleases, including Cas12 (Cpf1) and Cas13 (C2c2). Cas12a enables multiplex genome editing through its ability to process multiple guide RNAs (gRNAs) from a single transcript, supporting the simultaneous modification of polygenic traits—such as pyramiding disease-resistance alleles in soybean (*Glycine max*) (Sun et al. 2024). Meanwhile, Cas13d, when integrated with a polycistronic tRNA-gRNA (PTG) architecture, has demonstrated robust efficacy in targeting RNA viruses, offering a promising antiviral strategy in crops like potato (*Solanum tuberosum*) (Zhan et al. 2023). These advances provide a powerful and increasingly refined platform for crop genome engineering. Applying these tools to *Capsicum* could enable precise interrogation of stress-responsive regulatory networks and facilitate the tailored development of climate-resilient chili pepper cultivars.

Building on the transformative potential of CRISPR/Cas systems, the past decade has ushered in a wave of innovations that have expanded the precision and versatility of genome engineering. Breakthroughs such as base editing, prime editing, and the discovery of novel CRISPR-associated nucleases have significantly broadened the scope of gene functional analysis and accelerated the genetic improvement of crops (Fig. 1) (Das et al. 2022). Prime editing, which fuses a catalytically impaired Cas9 (dCas9) to a reverse transcriptase, allows programmable and precise nucleotide substitutions without requiring double-strand breaks or donor templates (Yan et al. 2024). This system holds the theoretical capacity to correct up to 89% of known pathogenic genetic variants and has been successfully deployed to engineer bacterial blight resistance in rice (*Oryza sativa*) by precisely rectifying disease-susceptible alleles (Gupta et al. 2023; Yan et al. 2024). In parallel, base editors, including cytosine base editors (CBEs) and adenine base editors (ABEs), facilitate irreversible single-nucleotide conversions, offering exceptional control over targeted mutagenesis with minimal genomic disruption (Pfeiffer and Stafforst 2023). These tools have enabled traits such as enhanced flavor profiles in tomato (*Solanum lycopersicum*) (Nizampatnam et al. 2024) and photoperiod sensitivity modulation in soybean through precise editing of *Flowering locus T* (*GmFT2a* and *GmFT4*), key regulator of flowering time (Cai et al. 2020).

Despite these technological advances, the application of CRISPR/Cas9 in *Capsicum* remains in its infancy, with only a limited number of successful cases documented to date (Table 2). Initial efforts have largely concentrated on fortifying disease resistance and enhancing tolerance to abiotic stress. Targeted editing of resistance (*R*) genes has reinforced immune responses against key pathogens, while precise modulation of stress-responsive signaling pathways has enhanced plant resilience under environmental extremes (Mishra et al. 2021; Vijay et al. 2023; Kalita et al. 2024). However, translating these strategies into robust, field-ready varieties remain challenging. The genetic complexity of *Capsicum*—characterized by extensive gene families, large and repetitive genomes, and in some species, polyploidy—complicates target identification and reduces editing precision. Moreover, technical limitations such as off-target mutations and recalcitrance to tissue culture and plant transformation hinder the scalability of genome editing platforms in this genus. Overcoming these barriers will require integrated approaches that combine advanced genome editing tools, genotype-independent transformation systems, and deeper functional insights into stress-regulatory networks in *Capsicum* species.

**Table 2:** Advances in chili pepper genome editing using CRISPR/Cas

Genotypes	Targeted gene	Gene function	Transformation method	Efficiency (%)	References
G4	*CaPDS*	Carotenoid biosynthesis	Cotyledon-biolistic	62.5	Bulle et al. (2024a)
Dempsey C15 Younggo 4	*CaPAD1*	Inducing parthenocarpy	Protoplasts-PEG-mediated transfection	7.2–11.3	Choi et al.(2024)
D21101, D21102, E21301, E21302, F21201, J21401	*CaMLO2*	Powdery mildew disease resistance	Protoplasts-PEG-mediated transfection	6.3–17.7	Park and Kim (2023)
CM334			Callus- *Agrobacterium tumefasciens*-mediated transformation	0.031–0.038	Park et al. (2021)
Dempsey				0.026–0.029	
CM334 Dempsey			Callus protoplasts-PEG-mediated transfection	11.3–17.6	Kim et al. (2020)
Arka Lohit	*CaERF28*	Anthracnose disease resistance	Hypocotyl- *Agrobacterium tumefasciens*-mediated transformation	70.37–74.28%	Mishra et al. (2021)

*CaPDS-C. annuum Phytoene Desaturase*; *CaPAD1- C. annuum Parental Advice-1*; *CaMLO2- C. annuum Mildew Locus O 2*; *CaERF28-C. annuum ethylene-responsive factor 28*

### Turning challenges into opportunities: practical advances and bottlenecks in chili pepper genome editing

While CRISPR/Cas technology holds tremendous promise for advancing chili pepper improvement, its widespread application remains constrained by several technical bottlenecks. These include limitations in vector design, suboptimal transformation and regeneration efficiencies, and the complex translation of genotypic edits into consistent phenotypic outcomes. Table 2 summarizes recent genome editing efforts in chili pepper, detailing target genes, enhanced genotypes, transformation protocols, editing efficiencies, and key trait improvements. The design of the CRISPR/Cas expression cassette—shaped by gRNA selection, promoter compatibility, and vector architecture—is a critical determinant of editing precision and efficiency. Multiple interdependent factors influence this step, including the choice and number of gRNAs, which is particularly relevant in polyploid *Capsicum* species (Haque et al. 2018). Among various promoter options, the *Arabidopsis thaliana* U6-26 (*AtU6-26*) promoter has emerged as a widely adopted element for gRNA expression, achieving an editing efficiency of 62.5% in *C. annuum Phytoene Desaturase* (*CaPDS*) gene (Bulle et al. 2024a). Beyond promoter selection, fine-tuning Cas protein expression can further enhance mutagenesis outcomes. Notably, Mishra et al. (2021) reported a 74.28% editing efficiency in *C. annuum ethylene-responsive factor 28* (*CaERF28*) using a single transcript unit (STU) system driven by a *Cauliflower Mosaic Virus 35S* (*CaMV 35S)* promoter to co-express sgRNA and *Streptococcus pyogenes* Cas9 (SpCas9). In addition engineering Cas coding sequences with nuclear localization signals (NLSs) and synthetic introns has been shown to enhance editing efficacy in other plants such as rice and *Arabidopsis* (Zhong et al. 2020; Grützner et al. 2021). Translating these innovations into *Capsicum* systems could markedly advance editing precision and phenotypic predictability.

In addition to vector architecture, the precision and efficiency of genome editing in chili pepper hinge on optimal guide RNA (gRNA) design. However, computationally optimized gRNAs often underperform in vivo due to context-specific biological constraints (Li et al. 2021). A range of bioinformatics tools, including CRISPR-P 2.0 (Lei et al. 2014), CHOPCHOP (Montague et al. 2014), and CRISPR Direct (Naito et al. 2015), facilitates gRNA design in *Capsicum*, incorporating assessments of on-target activity, off-target potential, and sequence context. Strategic mismatches within the seed region (8–12 nucleotides adjacent to the protospacer adjacent motif, PAM) can minimize off-target effects (Modrzejewski et al. 2020), while gRNAs with moderate guanine–cytosine (GC) content balance hybridization strength and specificity, as excessive GC pairing can exacerbate non-specific binding (Touzdjian Pinheiro Kohlrausch Távora et al. 2022). Beyond conventional (CRISPR/Cas9) editing, catalytically inactive Cas9 (dCas9) expands the functional scope of CRISPR tools through transcriptional regulation rather than DNA cleavage (Morgante et al. 2020). Though not yet implemented in *Capsicum*, dCas9-based systems enable CRISPR interference (CRISPRi) and activation (CRISPRa), modulating gene expression without altering genomic sequences (Fig. 1) (Gilbert et al. 2014). CRISPRi is most effective when gRNAs are targeted within 0–50 bp upstream of transcription start sites (TSSs) in open chromatin regions (Radzisheuskaya et al. 2016; Moradpour and Abdulah 2020). In CRISPRa, transcriptional activators are recruited via fusion proteins or engineered gRNA scaffolds. Systems such as dCas9–SunTag (Papikian et al. 2019), dCas9–TV (Xiong et al. 2021), dCasEV2.1 (Selma et al. 2019), and CRISPR-Act2.0 (Lowder et al. 2018), have progressively enhanced activation efficiency through multimerized effector domains and scaffold modularity. Together, these innovations underscore the centrality of gRNA design not only for targeting fidelity but also for functional recruitment in regulatory genome engineering (Moradpour and Abdulah 2020).

Multiplexed CRISPR systems, which employ multiple gRNAs to simultaneously target several loci concurrently, hold great promise for dissecting and engineering complex traits (Das et al. 2022). However, increasing the number of gRNA can compromise efficiency due to guide competition and limitations in the cellular processing machinery (Li et al. 2021). In a comparative study of the *C. annuum Mildew Locus O* 2 (*CaMLO2)* gene in the chili pepper cultivar ‘Dempsey,’ Kim et al. (2020) reported marked disparities in editing outcomes (Table 2): In protoplasts, sgRNA1 induced an 11.3% indel frequency in protoplasts, whereas sgRNA2 achieved only 0.5%. In callus tissue from ‘CM334,’ sgRNA1 reached 17.6% editing, while sgRNA2 yielded a negligible 0.2%. Notably, the CRISPR/LbCpf1 system produced the opposite trend: crRNA2 outperformed crRNA1, underscoring the critical influence of gRNA sequence, genome editing platform, and host genotype on editing outcomes (Kim et al. 2020). Supporting these findings, Bulle et al. (2024a) reported a 62.5% editing efficiency in *Capsicum* using two distinct gRNAs, further highlighting the importance of empirical, context-aware optimization (Table 2). Despite these advances, chili pepper remains highly recalcitrant to in vitro regeneration and stable transformation (Ahmad et al. 2021a, 2021b), a major bottleneck in CRISPR-based crop improvement. Consequently, transient systems such as protoplast transfection and *Agrobacterium rhizogenes*-mediated hairy root induction have gained traction as efficient platforms for construct validation (Carrijo et al. 2021; Choi et al. 2024), offering rapid editing workflows and higher throughput compared to traditional stable transformation.

Notably, Bulle et al. (2024a) and Mishra et al. (2021) successfully generated stable genome-edited chili pepper lines via particle bombardment and *Agrobacterium*-mediated transformation, respectively. In parallel, emerging CRISPR delivery platforms—such as nanoparticle-based carriers (Cunningham et al. 2018; Mahmoud and Dutt 2024; Hubbard et al. 2025), viral vector systems (Lee et al. 2024), and tissue culture-independent genome editing approaches (Quiroz et al. 2024; Kshetry et al. 2025; Hu and Liu 2025), are beginning to redefine plant transformation paradigms. Although these innovative systems have yet to be deployed in *Capsicum*, their future adaptation could significantly advance genome editing, especially in genotypes recalcitrant to conventional transformation. To further overcome regeneration bottlenecks, integrating developmental regulators such as BABY BOOM (*BBM*), WUSCHEL2 (*WUS2*), and plant growth-promoting factors like GROWTH-REGULATING FACTORS (*GRFs*) into transformation pipelines has emerged as a powerful strategy. In sweet pepper, *BBM* overexpression doubled transformation efficiency from 0.6 to 1.1% (Heidmann et al. 2011). More recent efforts employing GRF-INTERACTING FACTOR (*GIF*) chimeras or transcription factors such as PLETHORA3 (*PLT3*) and WUSCHEL-RELATED HOMEOBOX (*WOX*) have substantially enhanced regeneration efficiency across diverse crops (Youngstrom et al. 2025). The CRISPR-Combo system exemplifies this approach: in rice, co-activation of *BBM1* within the editing cassette markedly improved shoot regeneration and facilitated the recovery of edited lines (Pan et al. 2022). A parallel strategy in tomato, utilizing *GRF–GIF* chimeras, significantly reduced the time required to regenerate transgenic shoots (Swinnen et al. 2024).

Looking ahead, integrating CRISPR/Cas9 with high-resolution transcriptomic and metabolomic data offers a powerful framework for precision editing in *Capsicum*. Omics-driven studies in nightshade family crops have identified key regulators of osmotic balance, ROS detoxification, and hormonal signaling—pathways integral to stress resilience (Wang et al. 2019; Islam et al. 2023a; Morales-Merida et al. 2024). Translating these insights into chili pepper can inform the rational selection of candidate genes for genome engineering. TFs, in particular, represent strategic targets for multiplex editing, given their capacity to orchestrate entire gene regulatory networks (Zhang et al. 2021). This approach holds particular promise for improving complex traits such as capsaicinoid biosynthesis, drought tolerance, and postharvest longevity (Fig. 2). Coupled transcriptomic and metabolomic profiling can illuminate regulatory hierarchies and metabolic bottlenecks, enabling data-driven CRISPR designs (Patra et al. 2024; Fraser et al. 2020). However, critical challenges remain—including the scarcity of high-quality reference genomes across diverse *Capsicum* genotypes, limited transformation efficiencies, and the translational gap between omics insights and functional trait development (Vijay et al. 2023). Addressing these barriers will require interdisciplinary strategies that leverage systems biology and machine learning to prioritize editing targets and accelerate the development of climate-resilient, nutritionally enriched chili peppers.

### Engineering stress-adaptive transcriptional networks in chili pepper: from functional insights to CRISPR-driven precision breeding

As the convergence of multi-omics and functional genomics ushers in a new era of precision crop engineering, TFs have emerged as central molecular switches in orchestrating complex stress responses. These regulatory proteins function as rheostats, fine-tuning gene expression in response to diverse environmental stimuli, and are instrumental in shaping climate-resilient phenotypes. In chili pepper, TFs sit at the nexus of intricate signaling cascades, translating abiotic stress cues into coordinated transcriptional programs—positioning them as indispensable targets for stress-adaptive breeding (He et al. 2023) (Table 1). A compelling example is CaNAC035, a nucleus-localized NAC-domain TF that functions as a key activator of abiotic stress responses (Zhang et al. 2020b, 2024a). Its expression is rapidly induced by salinity, drought, temperature extremes, osmotic stress, and multiple phytohormones (Ou et al. 2024). Functional studies highlight its pivotal role in stress mitigation: silencing *CaNAC035* in pepper triggers oxidative damage, marked by elevated malondialdehyde (MDA) levels and ROS accumulation, whereas overexpression in *Arabidopsis* mitigated oxidative stress and enhances biomass accumulation and seed germination under stress conditions (Zhang et al. 2020b). Crucially, CaNAC035 is not an isolated regulator—it physically interacts with at least 18 stress-associated proteins, many of which are linked to defense responses and photosynthetic processes, underscoring its role as a central integrator of stress signaling and energy metabolism (Zhang et al. 2020b, 2024a) (Table 1). These multifaceted interactions position CaNAC035 as a regulatory hub within the stress-response network. In addition, CaNAC035 has been associated with changes in chlorophyll content, reflecting its impact on photosynthetic capacity under stress conditions (Zhang et al. 2020b, 2024a). However, comprehensive data on specific metabolite production regulated by CaNAC035 remain limited, precluding detailed profiling of its metabolic impacts. Leveraging its potential through CRISPR-mediated editing—via promoter modulation or targeted allelic variation—could facilitate the development of chili pepper cultivars with enhanced resilience to environmental extremes. This strategy also offers a scalable model for improving other solanaceous crops facing similar abiotic challenges.

Additionally NAC transcription factors in chili pepper further illustrate the functional versatility of this gene family. CaNAC46, for example, is transcriptionally activated by a wide array of abiotic stresses—including drought, salinity, and temperature extremes—as well as key hormonal signals such as abscisic acid (ABA), salicylic acid (SA), and methyl jasmonate (MeJA) (Jia et al. 2024; Ma et al. 2021) (Table 1). Overexpression of *CaNAC46* in *Arabidopsis* enhances root system architecture under stress, whereas its silencing in pepper leads to impaired growth and diminished ROS detoxification capacity (Ma et al. 2021). Similarly, CaNAC2c plays a dual role in abiotic and biotic stress adaptation, integrating heat stress signaling with jasmonate-mediated immunity and ROS-responsive defense mechanisms (Han et al. 2023; Ma et al. 2021). It activates the expression of *CaHSFA5* (*C. annuum Heat shock factor 5*) and interacts with stress-related proteins such as *CaHSP70* (*C. annuum Heat shock protein 70*) and *CaNAC029*, collectively enhancing both thermotolerance and pathogen resistance (Diao et al. 2018; Cai et al. 2021). While its role in stress adaptation is evident, current literature provides insufficient data to link CaNAC2c directly to specific metabolite production. Preliminary studies suggest possible influences on stress-related compounds, but detailed metabolic profiles are not yet available, warranting further investigation to elucidate its regulatory effects. In parallel, WRKY transcription factors function as pivotal regulators of immune responses in chili pepper. *CaWRKY1* is rapidly upregulated upon pathogen challenge, and its overexpression enhances hypersensitive responses against *Tobacco mosaic virus* (*TMV*) and *Pseudomonas syringae* pv. *tabaci*, whereas silencing increases susceptibility to *Xanthomonas axonopodis* (Oh SangKeun et al. 2008; Hussain et al. 2019, 2024) (Table 1). Despite these findings, specific metabolite production data regulated by CaWRKY1 are sparse, limiting a comprehensive understanding of its metabolic regulatory scope. Likewise, CaWRKY27 mediates resistance to *Ralstonia solanacearum* and confers tolerance to abiotic stresses through coordination of SA, JA, and ethylene signaling pathways (Dang et al. 2014, 2018). Additional members, such as CaWRKY01-10 and CaWRKY08-4, contribute to defense against *Phytophthora capsici* in both chili and tobacco (*Nicotiana tabacum*), suggesting conserved evolutionary roles across solanaceous species (Cheng et al. 2024) (Table 1). Its regulatory activity also extends to stress-related compounds, including MDA, hydrogen peroxide (H_2_O_2_), superoxide radicals, and chlorophyll content, indicating a role in maintaining photosynthetic stability under adverse conditions (Dang et al. 2014, 2018). However, detailed metabolite production profiles associated with CaWRKY27 are currently unavailable, highlighting the need for further research to clarify its metabolic contributions.

These TFs form a dynamic regulatory framework capable of integrating diverse environmental cues into tailored transcriptional responses. Realizing their full potential, however, hinges on the precise modulation of their activity—an increasingly attainable goal with the advent of CRISPR/Cas-based genome editing. Although CRISPR applications in chili pepper remain in their early stages, functional genomics in *Capsicum* provide compelling targets for intervention. One such example is CaSBP12, a member of the SBP-box TF family, identified as a negative regulator of salt tolerance (Zhang et al. 2020c). Silencing *CaSBP12* (*Squamosa-promoter binding protein 12*) in pepper markedly reduced ROS accumulation and improved ion homeostasis under salinity stress (Zhang et al. 2020c), highlighting its promise for gene knockout strategies. Recent innovations in CRISPR technologies extend beyond gene disruption to include promoter editing, cis-regulatory element targeting, and epigenomic modulation (Ali et al. 2025). These advanced tools can be strategically employed to enhance the expression of stress-inducible TFs such as CaNAC035, CaNAC2c, or CaWRKY27, or to repress negative regulators like CaSBP12, enabling the rational engineering of more resilient transcriptional networks in *Capsicum*. Proof-of-concept studies in other crops strongly support this strategy. In rice, CRISPR-mediated knockout of *OsNAC45* and *OsPUB67 (O. sativa U-box E3 ubiquitin ligase 67)* revealed their respective roles in salt and drought tolerance (Duan et al. 2016; Qin et al. 2020; Zhang et al. 2020d), while disruption of *OsPQT3 (O. sativa Paraquat Tolerance 3)*, a negative regulator of salt stress response, enhanced tolerance by mitigating oxidative damage (Alfatih et al. 2020). In soybean, targeted editing of *AITRs* (*ABA-induced transcription repressors*) and *GmMYB118* conferred improved resilience to salinity and drought (Du et al. 2018; Wang et al. 2021). Collectively, these findings validate the feasibility of engineering TF-centered regulatory networks to enhance stress adaptation. Extending this approach to chili pepper is both timely and strategically sound. Precision editing of *Capsicum*-specific transcription factors—via gene knockouts, gain-of-function alleles, or promoter rewiring—offers a path to sculpt context-responsive transcriptional programs tailored to increasingly erratic environmental conditions (Fig. 1). When integrated with omics-based regulatory maps, such strategies will enable targeted trait enhancement while preserving developmental homeostasis.

In summary, CaNAC035, CaNAC2c, CaWRKY1, and CaWRKY27 represent high-confidence targets for CRISPR-enabled precision breeding in chili pepper. Their central roles in integrating environmental signals and orchestrating downstream transcriptional responses position them as ideal entry points for next-generation crop engineering. To fully capitalize on their potential, future efforts must extend beyond functional characterization toward reprogramming transcriptional logic—establishing *Capsicum annuum* as a model system for climate-resilient crop design. By harnessing the power of transcriptional re-engineering, chili pepper can be transformed into a robust, high-performing crop capable of withstanding climate extremes, with direct implications for food security, farmer resilience, and sustainable agriculture.

## Conclusion and future perspectives

The convergence of CRISPR-based genome editing and transcriptional reprogramming heralds a paradigm shift in *Capsicum* improvement, positioning TFs as central levers for engineering climate resilience. No longer passive regulatory elements, TFs such as CaNAC46, CaWRKY27, and CaSBP12, have emerged as dynamic control hubs orchestrating stress adaptation, redox homeostasis, and metabolic reconfiguration. The precision afforded by CRISPR/Cas systems, augmented by advanced editors, such as base and prime editors, and regulatory modulators like CRISPR-Combo, enables the fine-tuning of these master switches, even within the challenging genomic terrain of polygenic and recalcitrant *Capsicum* genotypes. Yet, translating these molecular advances into field-ready cultivars demands more than technological prowess. It requires a concerted strategy that fuses high-throughput functional genomics, transcriptomics, metabolomics, and next-generation transformation platforms. Innovations such as the deployment of morphogenic regulators (e.g., BBM and GRF-GIF chimeras), optimized gRNA design algorithms, and tailored delivery systems are beginning to overcome long-standing barriers in *Capsicum* regeneration and editing efficiency. Looking ahead, the integration of multi-omics datasets with TF-targeted genome editing will unlock deeper insights into regulatory hierarchies governing stress plasticity. This systems-level understanding will be pivotal for the rational design of resilient phenotypes capable of withstanding fluctuating climatic extremes. Moreover, expanding the *Capsicum* pan-genome and leveraging machine learning to predict TF regulatory networks could dramatically accelerate trait discovery and gene prioritization. Ultimately, the strategic and precise editing of transcriptional regulators in *Capsicum* is not merely a tool; it is an evolutionary accelerant. By embedding resilience into the regulatory circuitry of chili pepper, this approach lays the foundation for a new era of climate-smart horticulture—one in which productivity, nutritional value, and environmental adaptability are no longer trade-offs, but co-optimized outcomes.

## Data Availability

The authors confirm that the data supporting the findings of this study are available within the article.
